# *trans*-Dichlorido­bis(3,4-dimethyl­pyridine)platinum(II)

**DOI:** 10.1107/S1600536808041597

**Published:** 2008-12-13

**Authors:** Alexander N. Chernyshev, Nadezhda A. Bokach, Youlia A. Izotova, Matti Haukka

**Affiliations:** a Department of Chemistry, St. Petersburg State University, 198504 Stary Petergof, Russian Federation; b Department of Chemistry, University of Joensuu, PO Box 111, FI-80101 Joensuu, Finland

## Abstract

In the title compound, *trans*-[PtCl_2_(C_7_H_9_N)_2_], the Pt^II^ atom is located on an inversion center and is coordinated by two 3,4-dimethyl­pyridine ligands and two chloride ligands, resulting in a typical slightly distorted square-planar geometry. The crystallographic inversion centre forces the value of the C—N—N—C torsion angle to be linear and the 3,4-dimethyl-pyridine ligands to be coplanar.

## Related literature

For related complexes see: Tessier & Rochon (1999 ▶); Eremenko *et al.* (1997 ▶); Shaver *et al.* (2000 ▶); Zordan *et al.* (2005 ▶); Rochon *et al.* (1996 ▶); Colamarino & Orioli (1975 ▶). For the geometry of the pyridine ligand, see: Bond & Davies (2002 ▶). For related literature, see: Orpen *et al.* (1989 ▶). 
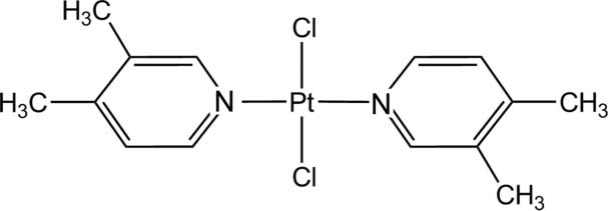


## Experimental

### 

#### Crystal data

[PtCl_2_(C_7_H_9_N)_2_]
*M* *_r_* = 480.29
Monoclinic, 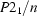

*a* = 7.9763 (5) Å
*b* = 7.1102 (3) Å
*c* = 13.3586 (7) Å
β = 98.247 (5)°
*V* = 749.77 (7) Å^3^
*Z* = 2
Mo *K*α radiation
μ = 9.70 mm^−1^
*T* = 120 (2) K
0.21 × 0.20 × 0.10 mm

#### Data collection

Nonius KappaCCD diffractometer
Absorption correction: multi-scan (*SADABS*; Sheldrick, 2003 ▶) *T*_min_ = 0.201, *T*_max_ = 0.381
17165 measured reflections
2177 independent reflections
1705 reflections with *I* > 2σ(*I*)
*R*_int_ = 0.033

#### Refinement

*R*[*F* ^2^ > 2σ(*F* ^2^)] = 0.015
*wR*(*F* ^2^) = 0.030
*S* = 1.08
2177 reflections
90 parameters
H-atom parameters constrained
Δρ_max_ = 0.67 e Å^−3^
Δρ_min_ = −0.78 e Å^−3^

### 

Data collection: *COLLECT* (Bruker–Nonius, 2004 ▶); cell refinement: *EVALCCD* (Duisenberg *et al.*, 2003 ▶); data reduction: *EVALCCD*; program(s) used to solve structure: *SHELXS97* (Sheldrick, 2008 ▶); program(s) used to refine structure: *SHELXL97* (Sheldrick, 2008 ▶); molecular graphics: *DIAMOND* (Brandenburg, 2006 ▶); software used to prepare material for publication: *SHELXL97*.

## Supplementary Material

Crystal structure: contains datablocks global, I. DOI: 10.1107/S1600536808041597/kj2108sup1.cif

Structure factors: contains datablocks I. DOI: 10.1107/S1600536808041597/kj2108Isup2.hkl

Additional supplementary materials:  crystallographic information; 3D view; checkCIF report

## Figures and Tables

**Table:** 

Pt1—N1	2.0148 (18)
Pt1—Cl1	2.2901 (6)

**Table:** 

N1—Pt1—Cl1	89.85 (6)

**Fig. 1.**
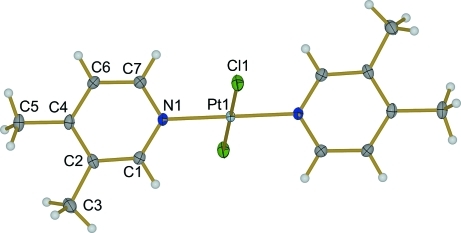
The molecular structure of the title molecule, with the atom-numbering scheme. Displacement ellipsoids are drawn at the 50% probability level.

## supplementary crystallographic information

### Comment

The complex *trans*-[PtCl_2_(C_7_H_9_N)_2_] has an inversion symmetry and the Pt^II^ atom is situated at an inversion center and it is coordinated by two 3,4-dimethylpyridine ligands and two chloro ligands and exhibit *trans* configuration. Such arrangement of ligands leads to the square planar geometry. In the coordination polyhedron, all angles are very close to the ideal value of 90°. The crystallographic inversion centre forces the value of the torsion angle C1—N1—N1^i^—C^i^ (symmetry operation i: -x, -y, -z) to be 180° and the the 3,4-dimethyl-pyridine ligands to be coplanar.

The geometry of 3,4-dimethylpyridine ligands resembles the geometry of the uncoordinated 3,4-dimethylpyridine, *i.e*. the C—C and C—N bond distances and angles in the coordinated 3,4-dimethylpyridine agree well with the expected value (Bond, Davies, 2002). The bond distance Pt–N (2.0148 (18) Å) is similar to the Pt—N bond lengths in other related compounds (Orpen *et al.*, 1989). The Pt—Cl bond lengths agree well with the reported values (See Table 2).

All *trans*-[PtCl_2_*L*_2_] complexes given in Table 2 have the same coordination environment as in the title compound. Indeed, they are square-planar and their pyridine rings lie in the same plane. The N—Pt—N and Cl—Pt—Cl angles in all observed compounds are equal to 180°, the angles N—Pt—Cl are very close to 90°.

### Experimental

3,4-dimethylpyridine (1 ml) was added to the powder of K_2_[PtCl_4_] (0.2 g) and the resulting mixture was heated to 150°C until the complete evaporation of the 3,4-dimethylpyridine. The resulting complex was recrystallized from CHCl_3_ (yield 92%). Crystals were obtained by slow evaporation of CHCl_3_ solution. Anal. calc. for C_14_H_18_N_2_Cl_2_Pt:*C*, 35.01; H, 3.78; N, 5.83%. Found: C, 35.30; H, 3.96; N, 5.54%.

### Refinement

Hydrogen atoms were positioned geometrically and constrained to ride on their parent atoms, with C—H = 0.95–0.98 Å, and *U*ĩso~ = 1.2–1.5 *U*~eq~(parent atom). The highest peak is located 0.87 Å from atom Pt1 and the deepest hole is located 0.83 Å from atom Pt1.

### Crystal data

**Table:**

[PtCl_2_(C_7_H_9_N)_2_]	*F*(000) = 456
*M**_r_* = 480.29	*D*_x_ = 2.127 Mg m^−^^3^
Monoclinic, *P*2_1_/*n*	Mo *K*α radiation, λ = 0.71073 Å
Hall symbol: -P 2yn	Cell parameters from 2339 reflections
*a* = 7.9763 (5) Å	θ = 1.0–20.0°
*b* = 7.1102 (3) Å	µ = 9.70 mm^−^^1^
*c* = 13.3586 (7) Å	*T* = 120 K
β = 98.247 (5)°	Block, pale yellow
*V* = 749.77 (7) Å^3^	0.21 × 0.20 × 0.10 mm
*Z* = 2	

### Data collection

**Table:**

Nonius KappaCCD diffractometer	2177 independent reflections
Radiation source: fine-focus sealed tube	1705 reflections with *I* > 2σ(*I*)
horizontally mounted graphite crystal	*R*_int_ = 0.033
Detector resolution: 9 pixels mm-1 pixels mm^-1^	θ_max_ = 30.0°, θ_min_ = 2.8°
φ scans and ω scans with κ offset	*h* = −11→10
Absorption correction: multi-scan (*SADABS*; Sheldrick, 2003)	*k* = −10→9
*T*_min_ = 0.201, *T*_max_ = 0.381	*l* = −18→18
17165 measured reflections	

### Refinement

**Table:**

Refinement on *F*^2^	Primary atom site location: structure-invariant direct methods
Least-squares matrix: full	Secondary atom site location: difference Fourier map
*R*[*F*^2^ > 2σ(*F*^2^)] = 0.015	Hydrogen site location: inferred from neighbouring sites
*wR*(*F*^2^) = 0.030	H-atom parameters constrained
*S* = 1.08	*w* = 1/[σ^2^(*F*_o_^2^) + (0.0064*P*)^2^ + 0.7739*P*] where *P* = (*F*_o_^2^ + 2*F*_c_^2^)/3
2177 reflections	(Δ/σ)_max_ < 0.001
90 parameters	Δρ_max_ = 0.67 e Å^−^^3^
0 restraints	Δρ_min_ = −0.78 e Å^−^^3^

### Special details

**Table:**

Geometry. All e.s.d.'s (except the e.s.d. in the dihedral angle between two l.s. planes) are estimated using the full covariance matrix. The cell e.s.d.'s are taken into account individually in the estimation of e.s.d.'s in distances, angles and torsion angles; correlations between e.s.d.'s in cell parameters are only used when they are defined by crystal symmetry. An approximate (isotropic) treatment of cell e.s.d.'s is used for estimating e.s.d.'s involving l.s. planes.
Refinement. Refinement of *F*^2^ against ALL reflections. The weighted *R*-factor *wR* and goodness of fit *S* are based on *F*^2^, conventional *R*-factors *R* are based on *F*, with *F* set to zero for negative *F*^2^. The threshold expression of *F*^2^ > σ(*F*^2^) is used only for calculating *R*-factors(gt) *etc*. and is not relevant to the choice of reflections for refinement. *R*-factors based on *F*^2^ are statistically about twice as large as those based on *F*, and *R*- factors based on ALL data will be even larger.

### Fractional atomic coordinates and isotropic or equivalent isotropic displacement parameters (Å^2^)

**Table:**

	*x*	*y*	*z*	*U*_iso_*/*U*_eq_	
Pt1	0.0000	0.0000	0.0000	0.01078 (3)	
Cl1	−0.12594 (8)	−0.27477 (8)	0.03883 (4)	0.01897 (11)	
N1	0.0259 (2)	0.0765 (3)	0.14663 (13)	0.0127 (4)	
C1	−0.0247 (3)	0.2464 (3)	0.17546 (16)	0.0143 (4)	
H1	−0.0716	0.3324	0.1246	0.017*	
C2	−0.0115 (3)	0.3015 (3)	0.27567 (16)	0.0141 (4)	
C3	−0.0729 (3)	0.4918 (3)	0.30137 (17)	0.0227 (5)	
H3A	−0.1179	0.5582	0.2390	0.034*	
H3B	0.0215	0.5637	0.3379	0.034*	
H3C	−0.1623	0.4781	0.3440	0.034*	
C4	0.0634 (3)	0.1776 (3)	0.35029 (16)	0.0138 (4)	
C5	0.0883 (3)	0.2318 (4)	0.45953 (16)	0.0188 (5)	
H5A	0.1668	0.3383	0.4702	0.028*	
H5B	0.1353	0.1249	0.5006	0.028*	
H5C	−0.0209	0.2676	0.4795	0.028*	
C6	0.1162 (3)	0.0044 (4)	0.31969 (15)	0.0161 (4)	
H6	0.1677	−0.0824	0.3688	0.019*	
C7	0.0948 (3)	−0.0435 (3)	0.21858 (17)	0.0163 (5)	
H7	0.1297	−0.1645	0.1994	0.020*	

### Atomic displacement parameters (Å^2^)

**Table:**

	*U*^11^	*U*^22^	*U*^33^	*U*^12^	*U*^13^	*U*^23^
Pt1	0.01278 (5)	0.01094 (5)	0.00822 (5)	0.00154 (6)	0.00017 (3)	−0.00158 (5)
Cl1	0.0265 (3)	0.0165 (2)	0.0140 (2)	−0.0048 (2)	0.0032 (2)	−0.0015 (2)
N1	0.0139 (9)	0.0141 (8)	0.0096 (8)	0.0001 (8)	0.0003 (7)	−0.0017 (7)
C1	0.0145 (10)	0.0156 (11)	0.0126 (9)	−0.0004 (9)	0.0014 (8)	0.0006 (8)
C2	0.0168 (11)	0.0133 (11)	0.0132 (10)	−0.0030 (9)	0.0050 (8)	−0.0033 (8)
C3	0.0370 (13)	0.0158 (10)	0.0165 (10)	0.0026 (13)	0.0077 (9)	−0.0002 (11)
C4	0.0122 (10)	0.0181 (11)	0.0111 (10)	−0.0041 (9)	0.0019 (8)	−0.0013 (8)
C5	0.0222 (12)	0.0232 (12)	0.0109 (10)	−0.0042 (10)	0.0018 (9)	−0.0026 (9)
C6	0.0168 (9)	0.0174 (10)	0.0131 (9)	0.0019 (11)	−0.0013 (7)	0.0011 (11)
C7	0.0166 (11)	0.0173 (12)	0.0147 (10)	0.0027 (8)	0.0008 (8)	−0.0010 (8)

### Geometric parameters (Å, °)

**Table:**

Pt1—N1^i^	2.0148 (18)	C3—H3B	0.9800
Pt1—N1	2.0148 (18)	C3—H3C	0.9800
Pt1—Cl1^i^	2.2901 (6)	C4—C6	1.382 (3)
Pt1—Cl1	2.2901 (6)	C4—C5	1.495 (3)
N1—C7	1.343 (3)	C5—H5A	0.9800
N1—C1	1.347 (3)	C5—H5B	0.9800
C1—C2	1.384 (3)	C5—H5C	0.9800
C1—H1	0.9500	C6—C7	1.380 (3)
C2—C4	1.398 (3)	C6—H6	0.9500
C2—C3	1.495 (3)	C7—H7	0.9500
C3—H3A	0.9800		
			
N1^i^—Pt1—N1	180.0	C2—C3—H3C	109.5
N1^i^—Pt1—Cl1^i^	89.85 (6)	H3A—C3—H3C	109.5
N1—Pt1—Cl1^i^	90.15 (6)	H3B—C3—H3C	109.5
N1^i^—Pt1—Cl1	90.15 (6)	C6—C4—C2	117.88 (19)
N1—Pt1—Cl1	89.85 (6)	C6—C4—C5	121.0 (2)
Cl1^i^—Pt1—Cl1	180.0	C2—C4—C5	121.1 (2)
C7—N1—C1	118.30 (19)	C4—C5—H5A	109.5
C7—N1—Pt1	119.91 (15)	C4—C5—H5B	109.5
C1—N1—Pt1	121.79 (15)	H5A—C5—H5B	109.5
N1—C1—C2	123.1 (2)	C4—C5—H5C	109.5
N1—C1—H1	118.5	H5A—C5—H5C	109.5
C2—C1—H1	118.5	H5B—C5—H5C	109.5
C1—C2—C4	118.5 (2)	C7—C6—C4	120.6 (2)
C1—C2—C3	119.8 (2)	C7—C6—H6	119.7
C4—C2—C3	121.78 (19)	C4—C6—H6	119.7
C2—C3—H3A	109.5	N1—C7—C6	121.6 (2)
C2—C3—H3B	109.5	N1—C7—H7	119.2
H3A—C3—H3B	109.5	C6—C7—H7	119.2

Symmetry codes: (i) −*x*, −*y*, −*z*.

### Table 2 Geometrical parameters (Å) for the trans-[PtCl_2_L_2_] (L = pyridine-type ligand) complexes.

**Table:**

L	Pt—N	Pt—Cl	N—Pt—Cl
4-picoline [1]	2.024 (5)	2.3046 (18)	90.16 (12)
N-nitroxyethylnicotinamide [2]	2.019 (8)	2.311 (3)	90.8 (2)
4-vinylpyridine [3]	2.021 (3)	2.3000 (9)	89.9 (8)
3-fluoropyridine [4]	2.0177 (20)	2.3013 (12)	89.86 (9)
3-chloropyridine [4]	2.015 (3)	2.3001 (8)	90.55 (8)
3-bromopyridine [4]	1.992 (6)	2.3106 (16)	90.40 (19)
3-iodopyridine [4]	2.019 (5)	2.303 (3)	89.7 (2)
2,6-bis(hydroxymethyl)pyridine [5]	2.040 (7)	2.306 (3)	90^m^
pyridine [6]	1.977 (2)	2.308 (3)	88.01 (6)

In all structures Pt atom is located on an inversion centre. ^m^ = Pt is on a mirror plane. [1] Tessier & Rochon (1999); [2] Eremenko *et al.* (1997); [3] Shaver *et al.* (2000); [4] Zordan *et al.* (2005); [5] Rochon *et al.* (1996); [6] Colamarino & Orioli (1975).
